# Lettuce (*Lactuca sativa*, variety Salanova) production in decoupled aquaponic systems: Same yield and similar quality as in conventional hydroponic systems but drastically reduced greenhouse gas emissions by saving inorganic fertilizer

**DOI:** 10.1371/journal.pone.0218368

**Published:** 2019-06-20

**Authors:** Hendrik Monsees, Johanna Suhl, Maurice Paul, Werner Kloas, Dennis Dannehl, Sven Würtz

**Affiliations:** 1 Leibniz-Institute of Freshwater Ecology and Inland Fisheries, Berlin, Germany; 2 Humboldt-Universität zu Berlin, Faculty of Life Sciences, Albrecht Daniel Thaer–Institute of Agricultural and Horticultural Sciences, Division Biosystems Engineering, Berlin, Germany; 3 Humboldt-Universität zu Berlin, Faculty of Life Sciences, Institute of Biology, Department of Endocrinology, Berlin, Germany; Ohio State University South Centers, UNITED STATES

## Abstract

Decoupled aquaponic systems have the potential to become one of the most effective sustainable production systems for the combined production of animal protein and plant crops. Here, recirculating aquaculture systems for fish production are combined with hydroponics for soilless plant production thereby recycling dissolved nutrients derived from metabolism of the fish. The aim of the present study was to characterize hydroponic lettuce production using conventional nutrient solution in comparison with decoupled aquaponics using the nutrient rich fish water as basis for the nutrient solution being supplemented by missing nutrients. In addition, one aquaponic treatment became disinfected in order to assess any occurring advantage of the aquaponics derived fish water. For evaluation the temperature, electrical conductivity, pH, and the mineral composition of the nutrient solution, as well as colony forming units in the fish water were monitored. Additionally, plant growth (fresh and dry weight, number and area of leaves) and quality parameters of lettuce leaves (nitrate, mineral content, phenolic compounds) were examined. Carbon sources and microorganisms derived from fish water seem to have neither beneficial nor detrimental effects on plant growth in this study. Except for some differences in the mineral content of the lettuce leaves, all other quality parameters were not significantly different. The use of aquaponic fish water saved 62.8% mineral fertilizer and fully substituted the required water for the nutrient solution in comparison to the control. Additionally, the reduced fertilizer demand using decoupled aquaponics can contribute to reduce greenhouse gas emissions of an annual lettuce production site per ha by 72% due to saving the energy for fertilizer production. This study clearly demonstrates the huge potential of the innovative approach of decoupled aquaponics to foster the transformation of our conventional agriculture towards sustainable production systems saving resources and minimizing emissions.

## 1 Introduction

The conventional production of animal protein for human consumption produces high amounts of liquid and organic waste (urine and faeces) that is regularly used to fertilize plant crops [1]. Due to its undirected application on agricultural landscapes this fertilization practise often results in eutrophication and pollution of the adjacent water bodies [1]. As Tilman, Balzer [2] reported, the global crop demand will increase by 100–110% from 2005 to 2050. The intensification of agriculture in turn, may have an immense negative impact on the environment [3]. As one approach to minimize the environmental impacts the so called “sustainable intensification” aims to increase the yield per unit area without increasing environmental burden [3, 4]. Sustainable intensification of crop production is hence a goal of the “Strategic Objective A” of the Food and Agriculture Organization of the United Nations (FAO). The FAO developed three intertwined pillars to reach sustainable intensification. Parts of them are the efficient use of resources, but also the protection of the environment by reducing pollution. One possibility to enforce these aims are aquaponic production systems as described below.

Within the last decade aquaculture has been the fastest growing sector in animal food production with average growth rates of 5.4% [5, 6]. Ongoing research and development provided the basis for a constant improvement of common aquaculture practises and increased the sustainability of aquaculture systems. In this context, recirculating aquaculture systems (RAS) have been developed to address several bottlenecks of open production systems [7]. Here, aquatic animals (mainly fish) are produced in closed systems with an integrated water treatment e.g. including biofilters for the conversion of ammonium (NH_4_^+^) to nitrate (NO_3_^-^) (nitrification) and mechanical filtration units (e.g. drum filter) for effective solids removal [7]. As a result of water treatment and low water consumption, nutrients (mainly nitrogen in the form of nitrate) and organic matter (often specified as dissolved organic carbon (DOC) and total organic carbon (TOC)) accumulate within these systems [8–10]. Consequently, if RAS are used for fish production, a recycling of RAS-derived nutrients for plant production should be promoted to close nutrient cycles and improve the resource efficiency. Nevertheless, it would be also possible to remove the nitrate via denitrification units [7], but here nitrogen would be lost, mainly in the form of N_2_.

Aquaponics is defined as the combination of fish rearing and hydroponic (soilless) plant production [11]. Water and accumulated nutrients from the fish unit, especially nitrogen, derived from the metabolism of the fish, but also from uneaten feed and organic debris [12], are used as fertilizer by the plants grown in hydroponics. In conventional aquaponics, also described as coupled aquaponic systems, the water flows from the fish unit to the plant unit and back [11, 13]. As such, compromises have to be made in terms of certain water parameters (e.g. pH, temperature, nutrient concentrations) either for the fish or for the plant production sites [14] leading to suboptimal conditions affecting adversely productivity of both, fish and plants. In addition to the well established coupled aquaponic systems, a decoupled approach may also be used [15], also referred to as double recirculating aquaponics system (DRAPS) [15, 16]. Here, fish are reared in a RAS and the plants are grown in a separate hydroponic system. The fish water from the RAS is directed to the reservoir of the hydroponic system on demand but not back to the RAS as it is the case in coupled aquaponics. Thereby the water parameters for each produced species (e.g. Nile tilapia *Oreochromis niloticus* and tomatoes, *Solanum lycopersicum*) can be optimized separately. As such, the nutrient concentrations and pH of the fish water can be adjusted to the specific demands of the growing crop. Thus, decoupled aquaponic systems maintain the benefits of aquaponics but simultaneously provide the opportunity to generate comparable productivities as in single conventional RAS and hydroponic units, respectively [15, 16]. Besides more theoretical considerations of decoupled aquaponics [17] or passing remarks as innovative approach [18–20], only a few empirical studies [15, 16, 21] are published yet. To our knowledge, only two publications comparing decoupled aquaponics to conventional hydroponics have been published so far [16, 21]. Delaide, Goddek [21] reported an increased lettuce growth of 39% in decoupled aquaponics compared to single hydroponic treatment and concluded that plant promoting effects like dissolved organic matter or growth-promoting rhizobacteria and/or fungi originating from the RAS water could be responsible for such an increased plant growth. On the contrary, Suhl, Dannehl [16] revealed a comparable tomato production in decoupled aquaponics and single hydroponics and confirmed that the productivity for tomatoes in DRAPS can reach the same level as in single hydroponics using optimum fertilizer but did not exceed the yield further as indicated by the results of Delaide, Goddek [21] concerning lettuce.

Therefore, the aim of this study was to evaluate the ability to produce butterhead lettuce **(***Lactuca sativa*, variety Salanova) in already established DRAPS as effectively as conventional hydroponics. The study was done with special emphasis on potential beneficial effects of dissolved organic matter and microorganisms derived from RAS and to highlight the resource efficiency of these aquaponic systems (in terms of water and nutrients). Finally the potential value of decoupled aquaponics for a sustainable intensification of agricultural production was evaluated.

## 2 Material and methods

### 2.1 Lettuce cultivation and experimental set-up

The experiments were conducted from 16^th^ March 2016 until 10^th^ May 2016. During this period green open butterhead lettuce Salanova (*Lactuca sativa* L., Rijk Zwaanm; De Lier, The Netherlands) cv. Descates RZ were grown hydroponically in a nutrient film technique system (NFT). The experiments were conducted in a 75 m^2^ compartment of an experimental Venlo-type greenhouse located in Berlin, Germany (52°46´82.806´´N, 13°29´88.909´´E). The ventilation was opened above 17°C and the target temperature for the floor level heating system was 10°C for night and 14°C for day. The mean climate values during the cultivation period are displayed in Table 1.

**Table 1:** Greenhouse climate during the experimental period of 7 weeks. The data represent the weekly mean values of temperature, relative humidity, carbon dioxide (CO_2_), and global radiation. Except global radiation, the mean values during daytime and night time are shown. For global radiation only the values for daytime are displayed.

Date		Temperature (°C)	Relative humidity (%)	Global radiation (W m^-2^)
**18.03.-27.03.2016**	day	14.78	54.47	174.47
	night	13.24	67.26	
**28.03.-03.04.2016**	day	13.36	39.64	277.36
	night	9.26	44.58	
**04.04.-10.04.2016**	day	16.59	46.85	255.66
	night	13.56	62.82	
**11.04.-17.04.2016**	day	15.55	58.43	186.32
	night	13.55	67.05	
**18.04.-24.04.2016**	day	15.60	42.22	359.82
	night	13.45	56.32	
**25.04.-01.05.2016**	day	15.88	43.03	329.52
	night	13.29	55.45	
**02.05.-10.05.2016**	day	21.80	40.33	414.72
	night	16.28	53.45	

**Fig 1:**
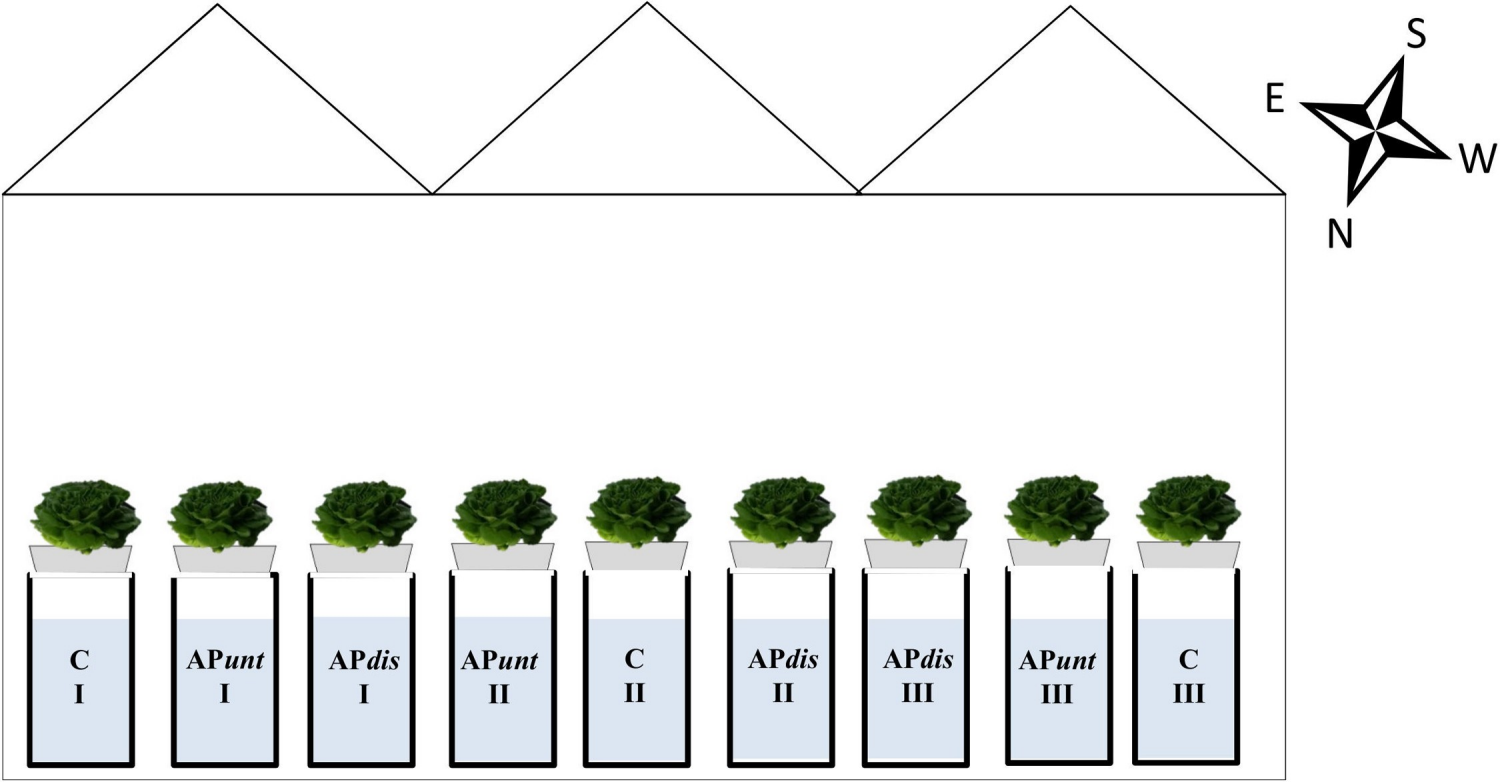
Schematic description of the arrangement of the experiments. C = control, fresh water based nutrient solution; AP*unt*—untreatend aquaponics, fish water based nutrient solution with supplemented nutrients; AP*dis*—like AP*unt*, but fish water was disinfected before use.

The lettuce seeds were sown in autoclaved cultivation soil where the plants grew for two weeks. After this period, the plants were transferred into rock wool cubes (7.5 cm x 7.5 cm, Cutilene; Tilburg, The Netherlands) using the cultivation soil to fix the plants and transplanted into high gullies on 18^th^ March 2016. A total of nine gullies (6 m length x 0.35 m width x 0.05 m high) with an inclination of 1% were installed in the greenhouse compartment. Ten lettuce plants were planted per gully with a distance of 50 cm apart (Fig 1). The space between the plants was covered with black and white plastic film to avoid algae formation. The distance between the gullies was around 0.5 m. At the end of each gully, a 300 L tank was installed as a reservoir for the nutrient solution. Conventional pumps (EHEIM universal 600, EHEIM GmbH & Co. KG; Deizisau, Germany) were used to pump the nutrient solution continuously (24 hd^-1^) to the end of each gully from where it was flowing back into the tank.

For the experiment, three different treatments were applied in triplicates (Fig 1). As such, nine gullies with ten lettuce plants each were used. In detail two nutrient solutions were prepared with fish waste water obtained from a recirculating aquaculture system rearing tilapia (see 2.1.2) and fertilizer addition. Afterwards, one of them was used directly (AP*unt* = Aquaponic untreated) for the experiments, whereas the other one was disinfected (AP*dis* = Aquaponic disinfected) before being used. For disinfection, the prepared nutrient solution was heated in a 150 L pot made from stainless steel to > 95°C for 15 min using a butane gas burner (3.6 kW) and three heating rods (stainless steel, 2 kW each). Afterwards the fish water was cooled down by a plate type heat exchanger. The preparation of the nutrient solution and disinfection procedure occurred at 16^th^ March 2016. Both treatments were compared to a control (C = Control), which was prepared with fresh tap and rain water (50:50, v/v, to reduce the amount of calcium) and mineral fertilizer.

#### 2.1.1 Adjustment of the nutrient solution

For optimal growth conditions, all nutrient solutions were adjusted with mineral fertilizer to an electrical conductivity (EC) of 2.2 dS m^-1^. The optimal nutrient solution for lettuce contained 200 mg N L^-1^, 62 mg P L^-1^, 150 mg K L^-1^, 210 mg Ca L^-1^, 50 mg Mg L^-1^, 70 mg S L^-1^. 2.5 mg Fe L^-1^, 0.62 mg Mn, L^-1^ 0.03 mg Mo, L^-1^ 0.09 mg Zn L^-1^, 0.50 mg Cu L^-1^ and 0.44 mg B L^-1^ as described by Hochmuth [22]. The mixture of rain and tap water (control) and fish water were analysed for its nutrient concentrations by inductively coupled plasma-optical emission spectrometry (ICP-OES) and continuous flow analysis (CFA) (see 2.2.1). Based on these results the required additions of mineral fertilizer were calculated to adjust the nutrient solution as given above. For preparing the nutrient solution, standard fertilizers were used: calcium ammonium nitrate (CAN; containing 1.1% ammonium), potassium nitrate (KNO_3_), mangnesium sulfate (MgSO_4_), potassium hydrogen phosphate (KH_2_PO_4_)_,_ and ready to use trace elements solution (YaraTera Tenso Cocktail, Yara UK Limited; N E Lincolnshire, United Kingdom). The adjustment of the pH value was done by supplementation of phosphoric acid to a pH of 5.8. The nutrient solutions were prepared in consecutive steps. In total, 750 L of nutrient solution (250 L per tank) for each treatment (control, AP*unt* and AP*dis*) was prepared, respectively (1 L stock solution per 100 L nutrient solution). Before the experiment started, a sample from each nutrient solution tank was taken and analysed by ICP-OES and CFA (see 2.1.1) to determine the nutrient composition. The results are expressed in mg L^-1^.

#### 2.1.2 Fish water

The fish water used to prepare the nutrient solution for both aquaponic treatments was taken from the aquaculture unit of the DRAPS located at the Leibniz-Institute of Freshwater Ecology and Inland Fisheries (IGB) in Berlin, Germany, and is described in detail by Monsees, Kloas [14]. The RAS unit was stocked with Nile tilapia (*Oreochromis niloticus*) at a mean stocking density of 90.6 kg m^-3^ in a total rearing volume of 5.1 m^3^ resulting in a total fish biomass of 462.1 kg. The pH of the water was kept at around 7.2 and controlled by addition of calcium hydroxide, the temperature was 25°C and the oxygen concentration remained always above 5.5 mg L^-1^. Fish were fed with floating pellets (ALLER SANA FLOAT, Emsland Aller Aqua GmbH; Golßen, Germany) containing 37% crude protein, 10% crude fat, 38.5% nitrogen-free extractive, 6% crude ash, 3.5% crude fibre and 17.9 MJ kg^-1^ digestible energy. The fish were fed at a moderate feeding rate of 0.65% of the bodyweight per day. The study was carried out in compliance with the German legislation as authorized by the Regional Office for Health and Social Affairs Berlin (permit #: ZH 114).

### 2.2 Sampling of the nutrient solutions

The samples for the different analyses were taken on a weekly basis (pH, EC, colony forming units (CFU)) or three times during the experiment (TOC, DOC). Except for CFU (see 2.2.4) the first samples were taken at 21^th^ March 2016 (3 days after planting) and marked as day or week 0, respectively. Samples for CFU were taken directly after disinfection (ad) (16^th^ March 2016). Samples for TOC and DOC were taken at 21^th^ March (week 0), in the 4^th^ and at the last day of the experiment (week 7).

#### 2.2.1 Nitrogen and nutrient analyses of process water and nutrient solutions

After collection all samples were frozen and stored in a freezer (-20°C) until further analysis of nitrogen and mineral nutrients.

To determine concentrations of ammonium nitrogen (NH_4_-N) and nitrate nitrogen (NO_3_-N) in process water and nutrient solutions the samples were filtrated through 0.45 μm cellulose acetate membrane filters (GE Healthcare, United Kingdom) before the analysis using continuous flow analysis (CFA; San++, Fa. SKALAR; Breda, The Netherlands). The used method is described in detail by Suhl, Dannehl [16] and outlined in detail in the supporting material (S1 File). The macronutrients (P, K, Ca, Mg, S, Na) and micronutrients (Fe, Mn, Mo, Zn, Cu, B) were analysed using inductively coupled plasma-optical emission spectrometry (ICP-OES, iCAP 6300Duo MFC, Fa. Thermo; Waltham, USA). After filtration, around 15 mL were used to flush the ICP-OES, and 45 mL were used for analysis. The measurement conditions (reference solution for calibration curves and wavelengths) for analysis of macronutrients are exactly described by Suhl, Dannehl [16] and Dannehl, Suhl [23] and are included in the supporting material (S2 File). Calibration curves for ICP-OES were made using micronutrient reference solutions with a concentration of 0–5 mg L^-1^ for Fe, Zn, Mn, Mo, Cu, and B.

#### 2.2.2 pH and electrical conductivity

The pH and EC of the nutrient solutions were measured weekly (HI9811-5, Hanna Instruments Inc.; Rhode Island, USA) and occurring changes of the pH values were adjusted using sodium hydroxide (NaOH).

#### 2.2.3 Total and dissolved organic carbon

For the analysis of total organic carbon (TOC) and dissolved organic carbon (DOC), two samples per nutrient solution were taken from each nutrient solution tank. For DOC analysis the samples were filtered through 0.45 μm cellulose acetate membrane filters (GE Healthcare, United Kingdom) being prepared by flushing with 20 mL distilled water and, subsequently with around 5 mL of the respective sample prior to sampling. The filtered DOC samples and the unfiltered samples for TOC analysis were subsequently acidified with 100 μL 2 M hydrochloric acid (HCl) and analysed using a TOC analyser following the NPOC-method according to the user’s manual (TOC LCPNF Analyzer, Shimadzu; Kyoto, Japan).

#### 2.2.4 Colony-forming units

For CFU analysis a dilution series (10^0^, 10^−1^, 10^−2^) was prepared using casein soybean flour peptone bullion (CASO Bullion, Carl Roth GmbH & Co. KG; Karlsruhe, Germany). Each dilution was plated on 100 mm petri dishes filled with CASO-agar (Carl Roth GmbH & Co. KG; Karlsruhe, Germany) and incubated for 48 h at room temperature (20°C). After incubation colonies were counted and CFU per mL was calculated. The whole work for CFU analysis was carried out in a microbiological safety cabinet (HeraSafe, Heraeus; Hanau, Germany).

### 2.3 Assessment of plant growth

At 10^th^ May 2016, the lettuce heads were harvested by cutting the aboveground organ directly above the rock wool cube. Immediately after harvesting, each lettuce head was weighed to determine the mean fresh weight (FW) in gram. Afterwards, the leaves of nine lettuce heads per treatment (3 per individual gully, Fig 1) were cut and counted. The results were expressed as number of leaves per plant. Subsequently, the counted leaves were used to measure the leaf area per plant in dm^2^ using an area meter (LI-3100 Area Meter; LICOR; Lincoln, NE, USA). The entire plant material was dried in a ventilated oven (Heraeus; Hanau, Germany) at 60°C until constant weight to determine the dry weight in gram, which was used to calculate the dry matter (DM) %.

### 2.4 Sampling and preparation for chemical analysis of lettuce

For determination of nitrogen, carbon, mineral elements, nitrate, as well as the concentration of poly-phenolic compounds, four replicates per treatment were used. Each replicate included a mix of three lettuce heads, which were randomly sampled. Afterwards, the 12 lettuce heads per treatment were quartered. Three quarters of different lettuce heads were pooled as one sample so that all mixed samples contained different lettuce heads. The samples were shock-frozen with liquid nitrogen and kept at -20°C until they were freeze-dried for 48 h (Christ Alpha 1–4, Christ; Osterode, Germany) and ground to a fine powder. Each freeze-dried sample was divided into four sub-samples, one for elemental analysis (EA), one for inductively coupled plasma-optical emission spectrometry (ICP-OES), one for nitrate analysis, and one for high performance liquid chromatography (HPLC).

#### 2.4.1 Nitrogen and carbon content as well as mineral elements in lettuce leaves

Nitrogen and carbon were determined according to DIN-ISO-10694 [24] and DIN-ISO-13878 [25] using an elemental analyser (vario MAX, Elementar Analysensysteme GmbH; Hanau, Germany). 0.3 g of the freeze dried samples was transferred into individual crucibles and catalytically combusted at 900°C with pure oxygen. To separate nitrogen and carbon, the combusted sample and a carrier gas (helium) passed through specific adsorption columns at a specific temperature of 830°C. Afterwards nitrogen and carbon content were analysed by a thermal conductivity detector and determined according to the different thermal conductivity of nitrogen and carbon. The analysis was carried out twice and the results were calculated by the standard reference material glutamic acid and expressed at % DM.

For analysing mineral elements in lettuce leaves, 0.498 to 0.502 gram of a freeze dried sample were weighted into special, de-ionised vessels and subsequently analysed by ICP-OES. The sample preparation and digestion in the microwave (MARS Xpress, CEM; North Carolina, USA) as well as the analysis by ICP-OES are described in detail by Dannehl, Huyskens-Keil [26] and in supporting information (S3 File). The backup ensued by double determination and the results were expressed as mg 100 g^-1^ FW.

#### 2.4.2 Nitrate content in lettuce leaves

Nitrate was analysed using a hand held reflectometer method (RQflex 10 plus, Merck KGaA; Darmstadt, Germany). For analysis 0.5 g of the powdered lettuce leaves were weighted into 50 mL volumetric flasks, filled up using distilled water and shaken for 20 min. Afterwards a barcode test strip was dipped into the sample solution for two seconds and the remaining solution was shaken off. During the reaction time of 60 seconds nitrate is reduced to nitrite. Nitrite reacts with an aromatic amine in the presence of an acid buffer to a diazonium salt. The diazonium salt in turn reacts with N-(1-Naphthyl)ethylenediamine to a red-violet azo dye. The quantity of the dye is measured by a reflectometer. The nitrate content was calculated and expressed in g kg FW^-1^.

#### 2.4.3 Analysis of phenolic compounds

For analysis of phenolic acids and flavonoids in lettuce leaves, 20 mg of the fine powder was extracted for 15 min in an ice water filled ultrasonic bath (Sonorex Super AG 102H, Bandelin electronic GmbH & Co. KG; Berlin, Germany) in 300 μL 70% methanol (pH 4.0, acetic acid). Afterwards, samples were centrifuged for 5 min at 4°C at 10000 rpm (Multifuge X1R, Thermo Fisher Scientific; Waltham MA, USA) and the supernatant was collected in a 2 mL Eppendorf tube. The pellet was extracted two times with 300 μL methanol (70%) for 10 min, respectively. The supernatants were concentrated in a vacuum concentrator evaporator (Savent SVC3000, Thermo Fisher Scientific; Waltham MA, USA) for around 2.5 h at 6 to 10 mbar. The resulting pellet was dissolved with 50% methanol and filled up to 1 mL. After shaking vigorously, 900 μL of the extract were filtered through a SpinX-Filter (Corning Costar Spin-X) upon centrifugation for 5 min at room temperature and 3000 rpm. Finally, the extract was transferred into 2 mL HPLC-Vials and stored at -20°C until analysis. 4-Methoxycinnamic acid (1 mM) and Apigenin-7-glucosid (0.5 mM) was used as internal standard for phenolic acids and flavonoids, respectively. For qualitative and quantitative analysis of the extracts the HPLC-system Ultimate 3000 (Thermo Fisher Scientific; Waltham MA, USA) was used as described by Förster, Ulrichs [27] and is outlined in supporting material (S4 File). Phenolic acids and flavonoids were detected at a wavelength of 290 nm and 370 nm. The identification of phenolic acids and flavonoids was carried out according to the molecular mass (MS measurement performed on selected samples), UV spectrum, retention time and standards. The results were expressed as mg 100 g^-1^ FM.

### 2.5 Calculation of fertilizer savings and greenhouse gas reduction

To calculate the fertilizer saving potential, the amounts of single fertilizer (macro nutrients) was calculated on a basis of g per L nutrient solution. The actual reduction was calculated referring to the control as 100%.

The reduction of greenhouse gases (expressed as CO_2_-equivalents (CO_2-eq_)) was calculated using the experimentally determined savings in inorganic fertilizers (aquaponic treatments) compared to the control (hydroponic treatment). The CO_2_-equivalents were thereby calculated using the open source database ProBas of the Federal Environment Agency of Germany [28]. The exact data used for calculation and an example how the CO_2_-equivalents were calculated for the respective fertilizers are listed in supporting material (S1 Table).

### 2.6 Statistical analysis

For statistical analysis of all evaluated parameters the SPSS package version 19.0 was used. As such, to analyse the differences between the nutrient solutions (nutrient composition, pH, EC, TOC and DOC) as well as the plant parameters (FW, No. of leaves, leaf area, DM, nutrient and nitrate concentration, and phenolic compound) univariate analysis of variance (ANOVA) was used after normal distribution was confirmed using Shapiro-Wilk test. For post hoc testing Tukey-HSD (homogeneous variances) or Dunnett-T3 (heterogeneous variances) test was used. Not normal distributed values were compared using Kruskal-Wallis test and Dunn-Bonferroni test. CFU data were evaluated using Kruskal-Wallis test for the initial measurement after disinfection (ad) test followed by a Friedman test for the analysis of the consecutive experimental weeks (week 0–7). Statistical analyses were carried out on a significance level of p < 0.05, where significant differences are indicated by different superscript small letters. When no superscript letters were inserted, no significant differences were detected. All data presented as mean values ± standard deviations of the respective samples.

## 3 Results

### 3.1 Plant yield and plant growth

The use of fish water, untreated or disinfected, resulted in similar yields as under conventional hydroponic conditions (Table 2). The lettuce heads reached a final fresh weight of 323.3 g (AP*unt = Aquaponic untreated*), 332.4 g (AP*dis = Aquaponic disinfected*) and 325.9 g (C = Control) after seven weeks of growth. Until harvest, the lettuce heads grown under control conditions formed 212.0 leaves. Lettuce heads in AP*dis* and AP*unt* formed 2 and 5.5 more leaves than the control, respectively. The formed leaf area was similar in all treatments and ranged between 66.7 dm^2^ (AP*unt*) to 67.5 dm^2^ (AP*dis*). The dry matter of lettuce grown under aquaponic conditions was 4.7%, 0.2% less than the control, though not significant.

**Table 2:** Growth parameters of butterhead lettuce grown in nutrient solution formulated with fresh water (control: 50:50, v/v fresh and rain water), fish water (untreated, AP*unt*) and disinfected fish water (AP*dis*). The fresh weight (FW), number of leaves (LN), leaf area (LA), and dry matter were compared and tested using univariate ANOVA. The data represent mean values of ten (FW) or three (LN, LA, DM) lettuce heads per repetition of each treatment.

Variant	Fresh weight (g)	Number of leaves	Leaf area (dm^2^ plant^-1^)	Dry matter (%)
**Control**	325.9 ± 53.6	212.0 ± 15.5	66.9 ± 5.7	4.9 ± 0.5
**AP*unt***	323.3 ± 44.5	214.1 ± 42.3	66.7 ± 6.8	4.7 ± 0.3
**AP*dis***	332.4 ± 55.5	217.5 ± 29.4	67.5 ± 7.2	4.7 ± 0.2

No significant differences (p < 0.05) were detected by post hoc Tukey HSD test (FW) or Dunnett-T3 test (DM) as well as Kruskal-Wallis test between the different treatments

### 3.2 Fertilizer and water savings

As evaluation criteria for improved environmental balance and to demonstrate the sustainability potential of the decoupled aquaponic system, the fertilizer savings for mixing the respective nutrient solutions were calculated (Table 3). The calculation was carried out based on single macro nutrients only. As such, the total fertilizer supply could be reduced by 62.8% using fish water to mix the nutrient solution for lettuce. CAN fertilizer was identified as the main fertilizer which was saved (84.6%), followed by KNO_3_ with a saving rate of 68.4%. Even the MgSO_4_ supply was reduced by 36.4%. The addition of KH_2_PO_4_ was only reduced by 7.4%, corresponding to 2 g per L stock solution. In consequence of the reduced fertilizer demand in the AP treatments, the calculated amount of greenhouse gas emissions (expressed as CO_2-eq_) by fertilizer manufacturing (incl. pre-process) could be reduced by 72.6% in comparison to the control. Additionally, in this setup, both aquaponic nutrient solutions were exclusively prepared with fish water, thus no additional freshwater was required (Table 3).

**Table 3:** Resource utilization (minerals, water) of butterhead lettuce grown in nutrient solution formulated with fresh water (control: 50:50, v/v fresh and rain water), fish water (AP*unt*) and disinfected fish water (AP*dis*).

Element	Unit^c^	Control^b^	AP*unt* / AP*dis*^d^	Reduction^e^ (%)	Control CO_2-eq_ (g L^-1^)^f^	AP*unt* / AP*dis*^d^ CO_2-eq_ (g L^-1^)^f^
**Fresh water^a^**	**L**	375	0	100		
**CAN**	**g L**^**-1**^	117	18	84.6	141.4	21.8
**KNO_3_**	**g L**^**-1**^	19	6	68.4	27.4	8.7
**MgSO_4_**	**g L**^**-1**^	44	28	36.4	13.0	8.3
**KH_2_PO_4_**	**g L**^**-1**^	27	25	7.4	16.9	15.6
**Total fertilizer supply/reduction/CO_2-eq_ emissions**	**g L**^**-1**^	**207**	**77**	**62.8**	**198.7**	**54.4**

^a^Total fresh water supply to all treatments; fresh water (50:50, v/v fresh and rain water)

^b^Control—fresh water prepared with mineral fertilizer; AP*unt* and AP*dis*—nutrient solution prepared with fish water and mineral fertilizer. AP*unt* was untreated and AP*dis* was disinfected nutrient solution.

^c^For fertilizer: g fertilizer use per L stock solution; 1 L stock solution is used to mix 100 L nutrient solution.

^d^Same values for AP*unt* and AP*dis*.

^e^Control is 100%.

^f^CO_2_-equivalent emission per L stock solution.

### 3.3 Nutrient composition of the used nutrient solution

The target nutrient concentrations for lettuce as recommended by Hochmuth [22] (see 2.1.1) were established here for N, K, Mg, and most micronutrients. Still, Ca content was slightly higher, and Fe concentration was lower than recommended. At the beginning of the experiment, the analysis of the nutrient solutions revealed comparable equal mineral N (N_min_ = NO_3_-N + NH_4_-N) concentrations (Table 4). Nevertheless, the NH_4_-N content was significantly higher in the control compared to both aquaponics treatments, whereas the NO_3_-N contents were comparable. Except K and the already mentioned N forms, nutrient solutions revealed significant differences for all nutrients determined. However, significant differences below 10% are likely irrelevant and will not be presented in detail here. Most obvious, P was almost 2-fold increased in the control, compared to both aquaponic treatments. Also, Ca was substantially lower in the control (287 mg L^-1^) than in both aquaponic treatments (319 and 322 mg L^-1^). Further, Cu, Mo, Mn, Mg and Zn differed between groups. Here, Mg, S, Mo, and Zn were significantly lower in the control than in both aquaponic treatments. Additionally, Na, Fe, Mn, Cu, and B were significant reduced in the control compared to at least one aquaponic treatment. Especially Fe and S were significantly decreased by 15-fold and 3.3-fold in the control.

**Table 4:** Nitrogen and mineral concentrations of the nutrient solution in mg L^-1^ at the beginning of the experiment used for control and both aquaponic treatments. The data represent the mean concentrations of nutrients in the three different nutrient solutions ± standard deviation measured after the nutrient solutions were mixed and completely prepared before the experiment started. The control was prepared with fresh and rain water (50:50, v/v), AP*unt* and AP*dis* were prepared with fish water. While AP*unt* was untreated, AP*dis* was disinfected. The data was compared and tested using univariate ANOVA and post hoc tests Tukey-HSD test or using Kruskal-Wallis (Cu) and Dunn-Bonferroni (Fe, Mo, B) test. Different superscript small letters indicate significant differences between the treatments (p < 0.05).

Element	Control	AP*unt*	AP*dis*
**NO_3_-N**	191.4 ± 8.2^a^	197.1 ± 10.5^a^	191.2 ± 22.9^a^
**NH_4_-N**	14.0 ± 0.9^c^	2.2 ± 0.01^a^	2.3 ± 0.01^b^
**N_min_^1^**	205.4 ± 9.1^a^	199.4 ± 10.5^a^	193.5 ± 23.0^a^
**P**	144.8 ± 0.5^b^	73.4 ± 0.9^a^	74.5 ± 0.4^a^
**K**	148.0 ± 1.5^a^	145.8 ± 1.5^a^	146.4 ± 1.9^a^
**Ca**	286.7 ± 3.7^a^	318.7 ± 3.6^b^	322.3 ± 1.0^b^
**Mg**	52.0 ± 0.5^a^	56.0 ± 0.3^b^	57.4 ± 0.5^c^
**S**	83.8 ± 1.6^a^	137.8 ± 1.3^b^	142.4 ± 0.9^c^
**Na**	55.2 ± 0.3^a^	55.6 ± 0.3^a^	57.0 ± 0.5^b^
**Fe**	0.001 ± 0.00^a^	0.01 ± 0.00^ab^	0.02 ± 0.00^b^
**Mn**	0.44 ± 0.00^a^	0.45 ± 0.01^b^	0.44 ± 0.00^a^
**Mo**	0.02 ± 0.00^a^	0.03 ± 0.00^b^	0.03 ± 0.00^b^
**Zn**	0.03 ± 0.01^a^	0.05 ± 0.01^b^	0.06 ± 0.01^b^
**Cu**	0.04 ± 0.04^ab^	0.03 ± 0.00^a^	0.06 ± 0.00^b^
**B**	0.24 ± 0.00^a^	0.28 ± 0.00^ab^	0.29 ± 0.00^b^

^1^N_min_ is the sum of NO_3_-N and NH_4_-N.

^a,b,c^ significant differences are indicated by different superscript small letters (p < 0.05)

### 3.4 pH value and electrical conductivity of the nutrient solution

**Fig 2:**
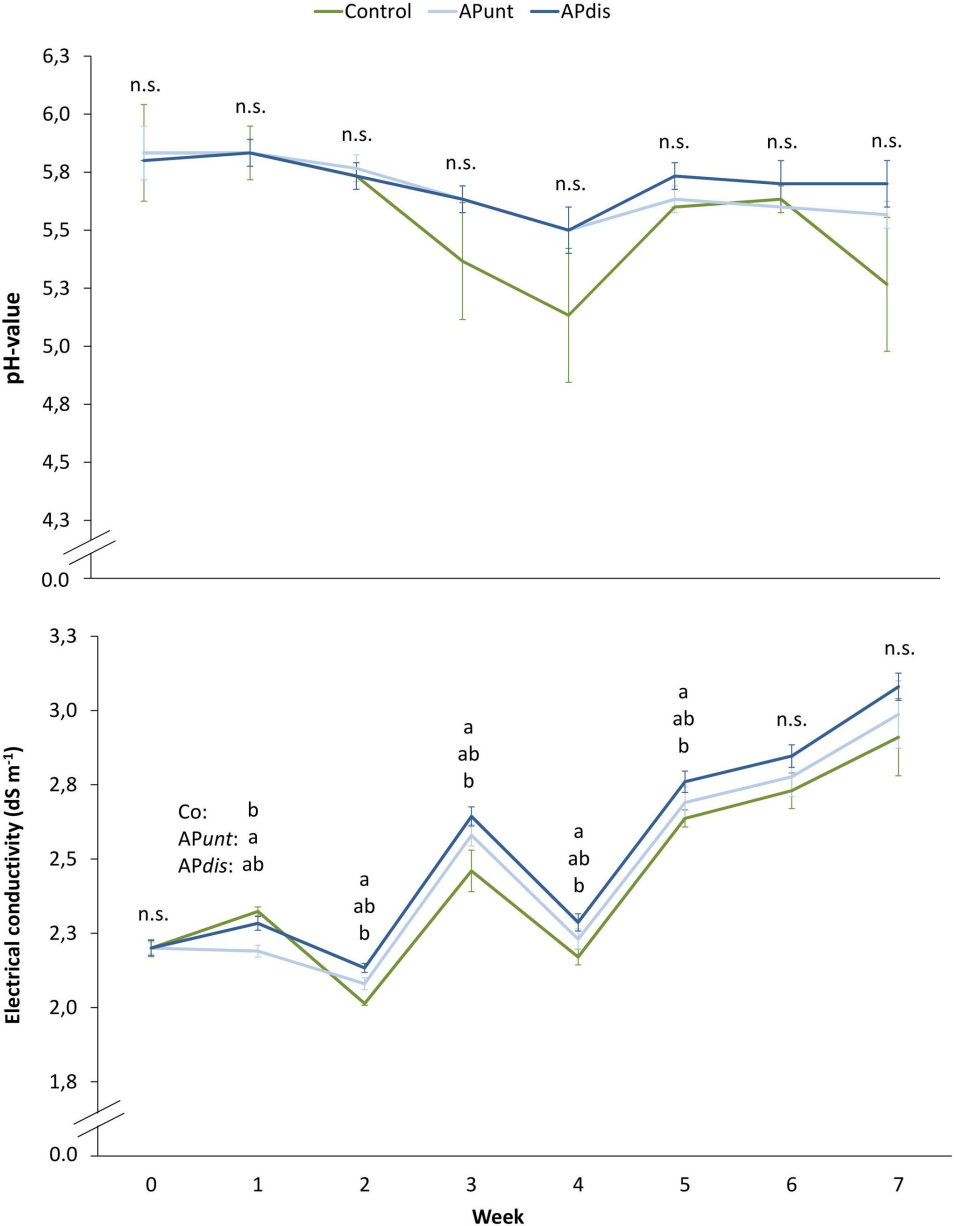
pH and electrical conductivity (EC) of the nutrient solutions formulated with fresh water (control: 50:50, v/v fresh and rain water), fish water (AP*unt*) and disinfected fish water (AP*dis*) over the experimental period of 8 weeks. The data represent mean values of three replicates ± standard deviation, measured once a week. The mean values were compared using univariate ANOVA and tested using post hoc tests Tukey-HSD (EC in week 3, 6, and 7) or compared using Kruskal-Wallis test (pH all and EC in week 0) and following post hoc Dunn-Bonferroni (p < 0.05). Different small letters indicate significant differences between the three different nutrient solutions at the respective time point and are listed from top to bottom: control, AP*unt*, and AP*dis*.

The weekly control of pH and electrical conductivity (EC) showed that the pH in the nutrient solution of the control started to drop down to 5.37 and 5.13 in the 3^rd^ and 4^th^ week, respectively (Fig 2). Still, differences to both aquaponic treatments were not significant. In the 5^th^ and 6^th^ week the pH levelled again at around 6 before it decreased again in week 7. However, the pH of the nutrient solution based on fish water was relative uniform and constant and varied only between 5.5 and 5.8.

In contrast, the EC level of all nutrient solutions varied stronger than the pH, but the development of the EC over time was similar in all treatments (Fig 2). As such, a more or less continuous increasing EC value from the beginning of the experiment (2.2 dS m^-1^) to the 7^th^ week (around 3.0 dS m^-1^) was observed. Nevertheless, the differences between the different treatments were significant from the 1^st^ to the 5^th^ week. The EC in AP*dis* was always significantly higher than in the control, except in week 1. The EC value of both aquaponic treatments did not differ over the experimental period. With an increasing standard deviation, the EC values did not differ significantly from each other in the last two weeks of the experiment.

### 3.5 Total and dissolved organic carbon

The measurement of TOC and DOC showed significant differences between the different nutrient solutions (Table 5). With the use of fish water, the aquaponic treatments contained concentrations at the beginning of the experiment for TOC 3.4-fold (AP*dis*) and 3.5-fold (AP*unt*) and for DOC 3.8-fold (AP*dis*) and 4.1-fold (AP*unt*), compared to the control. In the following weeks the differences decreased down to 2.9-fold (AP*unt*) and 3.0-fold (AP*dis*) for TOC and 3.2-fold (AP*unt*) and 3.4-fold (AP*dis*) for DOC, respectively. The disinfected nutrient solution (AP*dis*) had a significantly reduced DOC content compared to the untreated aquaponics (AP*unt*) at the beginning of the experiment. However, in the following weeks, the values dropped and were not significantly different any more.

**Table 5:** Total (TOC) and dissolved organic carbon (DOC) in the different nutrient solutions. The values represent mean values of three replicates ± standard deviation. Different superscript small letters indicate significant differences between the three treatments within one measuring week. The mean values were compared using univariate ANOVA and differences were tested using Dunnett-T3 test (p < 0.05). The control was prepared with fresh and rain water (50:50, v/v), AP*unt* and AP*dis* were prepared with fish water. While AP*unt* was untreated, AP*dis* was disinfected.

Week	Parameter	Control [mg L^-1^]	AP*unt* [mg L^-1^]	AP*dis* [mg L^-1^]
**0**	**TOC**	7.4 ± 0.85^a^	26.1 ± 0.44^b^	25.5 ± 0.59^b^
	**DOC**	5.9 ± 0.08^a^	24.3 ± 0.15^c^	22.6 ± 0.15^b^
**4**	**TOC**	8.0 ± 0.93^a^	23.1 ± 0.80^b^	24.2 ± 1.72^b^
	**DOC**	7.0 ± 0.87^a^	22.1 ± 0.94^b^	22.7 ± 1.17^b^
**7**	**TOC**	8.6 ± 0.97^a^	27.6 ± 2.25^b^	28.4 ± 0.54^b^
	**DOC**	8.1 ± 0.89^a^	26.8 ± 2.36^b^	27.9 ± 0.55^b^

^a,b,c^ significant differences are indicated by different superscript small letters (p < 0.05)

### 3.6 Colony-forming units content in the nutrient solutions

**Fig 3:**
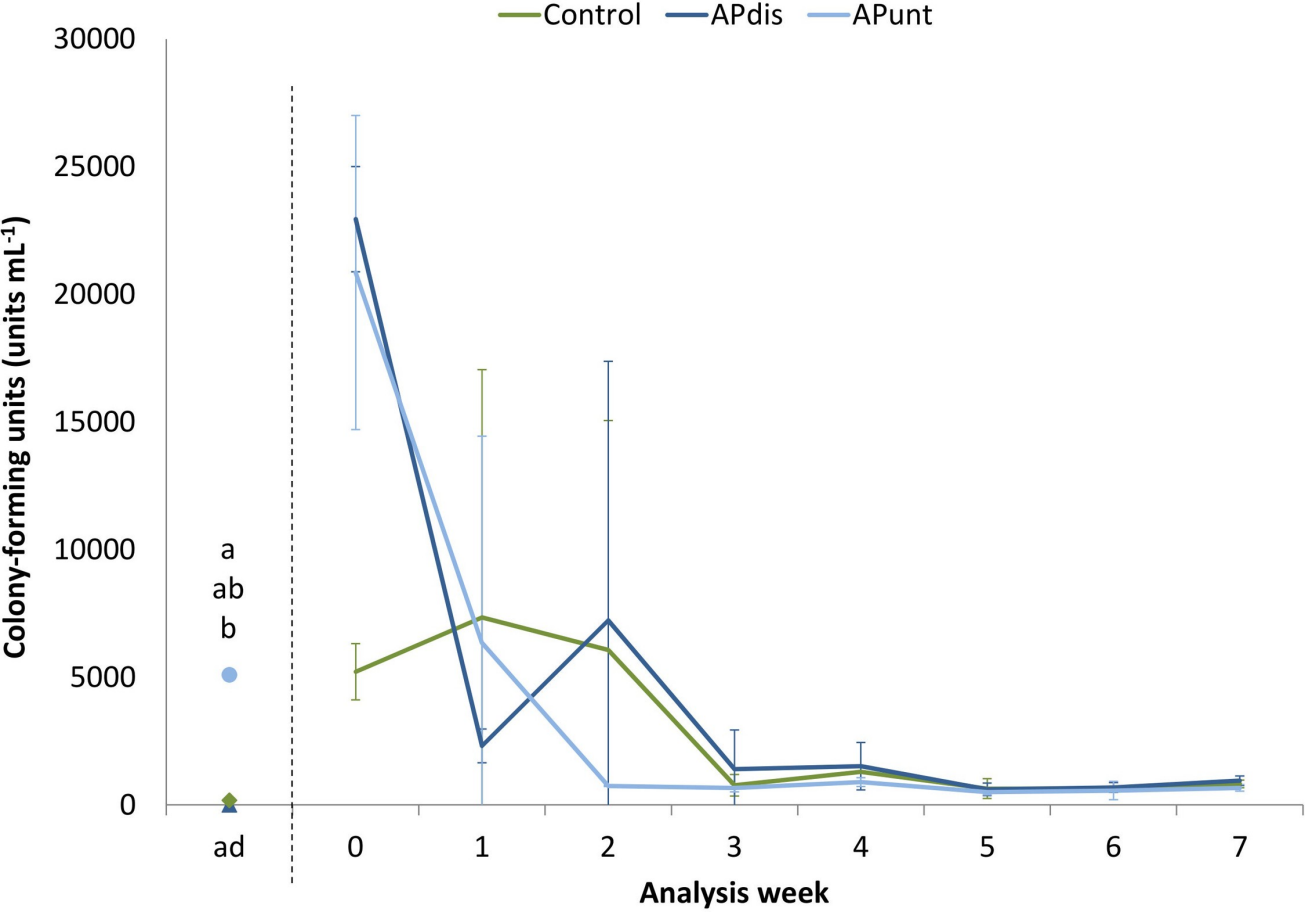
Content of colony-forming units (CFU) of the nutrient solution formulated with fresh water (control: 50:50, v/v fresh and rain water), fish water (AP*unt*) and disinfected fish water (AP*dis*) over the experimental period of 7 weeks. The data represent mean values of three repetitions per treatment ± standard deviation. Mean values were evaluated using Kruskal-Wallis test for the time after disinfection (ad) and by the Friedman test for the analysis of the consecutive experimental weeks (week 0–7) at a level of p < 0.05. Significant differences are indicated by different small letters and listed from top to bottom in the following range: AP*unt*, control, and AP*dis*.

No CFU were detected in AP*dis* after disinfection (Fig 3). The control started with an average CFU content of 177 colonies per mL^-1^ and contained significant more CFU than AP*dis* but significant less than the untreated fish water based nutrient solution (5097 CFU mL^-1^). From the day of disinfection until the first measurement on 21^st^ March 2016 (week 0, 5 days after disinfection, 3 days after planting; see 2.1), the CFU in AP*unt* and AP*dis* increased rapidly. In the following two weeks the CFU in both aquaponic treatments dropped down, but varied strongly between the replicates, as revealed by a high standard deviation. In the 3^rd^ and 4^th^ week the nutrient solutions of all treatments dropped to a relatively stable level of around 662 (AP*unt*) to 1518 (AP*dis*) CFU mL^-1^. From the 5^th^ week until the end of the experiment the CFU decreased further and ranged between 502 (AP*unt*) and 949 (AP*dis*) in all treatments. The Friedman test did not indicate significant differences between the treatments.

### 3.7 Nitrate, nitrogen, carbon, and mineral content in lettuce leaves

Lettuce leaves from the three treatment groups revealed significantly different contents of Ca, Mg, S, and Na (Table 6) on a dry mater basis as analysed by ICP-OES analysis of macro- and micro elements. Here, the Ca and Mg was significantly different in-between both aquaponics treatment but not to the control. Both were increased in the untreated aquaponics treatment (Ca = 9.8% and Mg = 10.7%). The content of S was significantly decreased (7.1%) in the conventional nutrient solution compared to AP*dis*. Also, Na was lower by 18.5% and thus significantly less in AP*unt* compared to the control. The amount of Fe was not significantly different between the treatments.

**Table 6:** Effect of fresh water (control, prepared using fresh and rain water (50:50, v/v), untreated fish water (AP*unt*) and disinfected fish water (AP*dis*) based nutrient solution on nitrate and mineral content in butterhead lettuce. The data represent mean values on dry weight matter (DM) or fresh weight basis (FW, only for NO_3_) ± standard deviation of four mix samples taken randomly from respective three lettuce heads. Mean values were compared using univariate ANOVA and following tested using post hoc Tukey-HSD test or compared by Kruskal-Wallis test (P) and tested using Dunn-Bonferroni test (Mg, Na) (p < 0.05). Significant differences are indicated by different superscript small letters.

Element	Unit	Control	Ap*unt*	AP*dis*
NO_3_	**mg kg**^**-1**^ **FW**	5896.3 ± 453.7^a^	5795.3 ± 456.3^a^	5502.1 ± 367.7^a^
**C**	**%DM**	42.25 ± 0.44^a^	42.48 ± 0.59^a^	42.0 ± 0.34^a^
**N**	**%DM**	4.12 ± 0.19^a^	4.29 ± 0.12^a^	4.27 ± 0.09^a^
**P**	**%DM**	0.71 ± 0.03^a^	0.73 ± 0.02^a^	0.73 ± 0.03^a^
**K**	**%DM**	6.33 ± 0.21^a^	5.94 ± 0.16^a^	6.29 ± 0.32^a^
**Ca**	**%DM**	1.79 ± 0.19^ab^	1.91 ± 0.06^b^	1.74 ± 0.06^a^
**Mg**	**%DM**	0.28 ± 0.03^ab^	0.31 ± 0.00^b^	0.28 ± 0.01^a^
**S**	**%DM**	0.26 ± 0.01^a^	0.27 ± 0.01^ab^	0.28 ± 0.01^b^
**Na**	**%DM**	0.27 ± 0.02^b^	0.22 ± 0.01^a^	0.21 ± 0.01^ab^
**Fe**	**%DM**	0.59 ± 0.06^a^	0.60 ± 0.10^a^	0.57 ± 0.02^a^

^a,b,c^ significant differences are indicated by different superscript small letters (p < 0.05)

### 3.8 Phenolic acids and flavonoids in lettuce leaves

The analysis showed that the investigated green butterhead lettuce contained mainly dicaffeolyltartaric (between 2.45 and 3.44 mg 100g^-1^ FW) (Table 7). The other phenolic acids did not follow a consistent range. The lowest concentrations were detected for coumaroylquinic acid ranging between 0.028 mg 100g^-1^ FW in control lettuce and 0.038 mg 100g^-1^ FW in AP*unt*. The content of the different phenolic acids, as well as the analysed flavonoid quercetin-3-O-(6´´-O-malonyl) did not differ significantly.

**Table 7:** Concentrations of phenolic acids and flavonoid glycosides in mg per 100 g fresh weight in butterhead lettuce caused by different nutrient solutions. The nutrient solutions for control were prepared with fresh and rain water (50:50, v/v) and for AP*unt* and AP*dis* with fish water. The latter were untreated for AP*unt* and disinfected for APdis. The data represent mean values of four lettuce heads samples, mixed in turn of three different lettuce heads ± standard deviation. No significant differences (p < 0.05) were analysed using univariate ANOVA and Tukey-HSD test (caffeoyltartaric acid, caffeoylquinic acid, and caffeoylmalic acid) or using Kruskal-Wallis test.

Metabolite	Control	AP*unt*	AP*dis*
***Phenolic acids***			
** caffeoyltartaric acid (CTA)**	0.97 ± 0.23	1.22 ± 0.34	1.29 ± 0.29
** caffeoylquinic acid (chlorogenic acid)**	0.93 ± 0.15	1.08 ± 0.24	1.12 ± 0.24
** coumaroylquinic acid**	0.028 ± 0.005	0.033 ± 0.013	0.038 ± 0.010
** caffeoylmalic acid (CMA)**	1.07 ± 0.22	1.13 ± 0.36	1.34 ± 0.36
** dicaffeoyltartaric acid (chicoric acid)**	2.45 ± 0.58	3.26 ± 0.74	3.44 ± 0.75
***Flavonoid***			
** quercetin-3-O-(6´´malonyl)glucoside (Q3MG)**	0.25 ± 0.03	0.33 ± 0.07^a^	0.36 ± 0.05

## 4 Discussion

### 4.1 Plant yield and plant growth

As one of the most important and obvious results of the present study, the fresh weight (FW) of butterhead lettuce did not differ significantly between both aquaponics treatments and the conventional hydroponic control (Table 2). Consequently, a similar number of leaves, leaf area and dry matter content of all lettuce heads were observed. These results are not surprising as nutrient solutions had comparable nutrient concentrations. As a logical consequence, the growth of plants should be comparable. As hypothesized by Delaide, Goddek [21], RAS derived carbon sources or microorganisms might have beneficial effects on plant growth cannot be supported in the present study. Nevertheless, it was found that decoupled aquaponics are as effective as conventional hydroponics but using around 63% less mineral fertilizer and 100% less fresh water for lettuce production (Table 3). Similar was found by [16, 29] who documented no significant differences in tomato yield between conventional hydroponics and supplemented aquaponics. In contrast, Delaide, Goddek [21] reported that the supplementation of fish water with mineral fertilizer increased the FW of lettuce by nearly 40%. Delaide, Goddek [21] concluded that substances with plant-promoting effects, like humic-like and protein-like dissolved organic matter (DOM) or rhizobacteria and/or fungi may have enhanced plant growth. According to this hypothesis, the present study should have resulted in differences between conventional and aquaponic treatments. As such, the growth-promoting effects of DOC would have increased the growth in untreated and disinfected aquaponics (AP*unt* und AP*dis*) compared to the control. Also, growth stimulation due to beneficial bacteria or fungi would have resulted in differences between AP*unt* and the control and potentially also between both aquaponic treatments. Adani, Genevini [30] found beneficial effects of humic acids on plant growth in hydroponics. As reported, humic acids added to hydroponics may have different effects on plant growth, e.g. an positive effect on shoot:root ratio [30]. The DOC determined in the waste water of RAS includes humic substances [31]. Humic substances are highly complex substances containing humid acids as extractable fractions [32] and the accumulation of humic substances in RAS depends on the feed input [9, 31] and/or the fresh water exchange rate [31]. Therefore, the composition of humic substances may vary and might be different from RAS to RAS, and study to study. As such, the question on how the DOC, the produced fish species and/or the composition of the fish feed might improve the above ground biomass of lettuce can result in divergent findings. Additionally, the current approach used a static nutrient supply as routinely used in lettuce production. For tomato or cucumber culture, a discontinuous refilling of the nutrient reservoir is necessary due to the extended culture period of these crops. This could eventually also result in different growth patterns such as improved growth in the aquaponic treatment compared the conventional hydroponic production since the exposure time of RAS derived microorganisms and organic carbon sources are prolonged.

### 4.2 Fertilizer savings and fertilizer use efficiency

Around 63% less mineral fertilizer was used in the aquaponic treatments compared to the hydroponic control (Table 3). Due to the nutrient composition of the fish water, especially CAN fertilizer addition was reduced (-84.6%) by decoupled aquaponic treatments. In contrast, the lowest reduction was realised with the phosphorus fertilizer (KH_2_PO_4_), which resulted in a reduction of 7.4%. Fertilizer production is an energy demanding process. As Jenssen and Kongshaug [33] reported, approximately 1.2% of the world´s energy consumption is assigned to fertilizer production which is responsible for 1.2% of the world´s greenhouse gas (GHG) emissions. GHG emissions originate e.g. from the manufacturing processes of nitrogen-containing fertilizer or from fossil fuel utilization for the production and the transport of raw material [34]. As a consequence, by reducing fertilizer use and decreasing fertilizer production, GHG emissions could be reduced substantially. Arguably, this is only the case if used as add-on for pre-existing or newly planned RAS, which primarily serve the purpose of satisfying an increasing demand for animal protein. Aquaponic systems could, due to the increased circularity and the recycling of nutrients, substantially reduce the demand for inorganic fertilizer, and thus lower GHG emissions compared to conventional hydroponic systems. However, considering a theoretical global dietary shift in the future towards plant based diets, one could argue that the energy requirements for e.g. building and operating a RAS, feed production, etc. will release the same or even higher GHG emissions compared to the production of inorganic fertilizer. Here, a RAS would primarily serve the purpose of nitrogen production.

Due to the large variety of fertilizers the direct calculation of GHG emissions from fertilizer production is difficult [34]. According to the calculation (see supporting material S1 Table) using ProBas database [28], the amount of CO_2-eq_ produced by 1 L stock solution was reduced by 72.6% using fish water to prepare the nutrient solution (Table 3). Considering a lettuce yield of 41 kg m^-2^ year ^-1^ and a water use of 20 L kg^-1^ year^-1^ [35] the total nutrient solution demand per year would be 8200 m^3^ per ha (i.e. 82 m³ fertilizer stock solution, compare Table 3). Extrapolated to an annual lettuce production in a one hectare greenhouse, the emitted GHG (manufacturing) would amount 16.3 t CO_2-eq_ for the conventional hydroponic production (control). In contrast, using nutrient enriched fish water could reduce this amount to only 4.5 t CO_2-eq_ (-72%) as shown in the present study.

### 4.3 Nutrient management in decoupled aquaponics

The process water for mixing the nutrient solutions for the aquaponics treatments was used directly from the RAS without prior dilution to reflect realistic, practical conditions. Here, it was possible to adjust most nutrients to nearly similar concentrations (Table 4). Nevertheless, some nutrients differed due to major differences in the aquaponic RAS and the tap/rain-water mixture as well as the standard fertilizer used here.

Similar to the finding of Delaide, Goddek [21], several elements were decreased, most prominent NH_4_^+^ (but not N_min_), P (2-fold) or increased Ca, S, Fe, Zn in the aquaponics compared to the control. Nitrogen is a decisive nutrient for plant growth [36]. Therefore, the focus for mixing the nutrient solution in the present study was to align N in the different treatments, which was only possible with regard to the total N. The nearly 2-fold higher P content in the control can be explained by the necessity of a much higher addition of phosphoric acid due to the higher alkalinity of the tap water to drop the pH to 5.8. In general, RAS process water often has a decreased alkalinity due to nitrification processes in the biofilter (release of protons) and due to an increased input of CO_2_ (respiratory end-product of fish and microorganisms). Nitric acid which is commonly used to lower the pH [37], would have increased the nitrogen content, which was undesirable. As a consequence of the levelling of N, the Ca content in all treatments was higher than recommended by Hochmuth [22] because it was added in combination with nitrate as calcium-ammonium-nitrate (CAN) fertilizer. The S content was 1.6-fold higher in both aquaponic treatments as a result of Mg equilibration by MgSO_4_. The significant differences between both aquaponic treatments were minor, though partially significant. Higher values upon disinfection (NH_4_-N, Mg, S, Na, and Cu) may be explained by the increased evaporation of water.

The interactions of soluble nutrients in the root zone itself can have effects on nutrient uptake by plants. Such interactions between nutrients are diversified [38]: While an increasing amount of specific nutrients, for example reported for S [39] and Zn [40], results in an increased accumulation of these nutrients in plants, other nutrients can have synergetic and antagonistic effects [38]. However, the influence of divergent nutrient solutions, without being more specific, resulted in a different nutrient content of the plants, not only between the control and at least one aquaponic treatment, but also between both aquaponic treatments (Table 6). The mineral content in all treatment groups showed no obvious deficiencies compared to the ranges reported by Hochmuth, Maynard [41]. Thus, significant differences in the nutrient concentrations of the different treatment groups had obviously no negative effects on the plant growth.

Due to the highly complex interactions of ions and the differences in the nutrient solutions, slightly different concentrations in the crop may occur but did ultimately not affect the plant growth here. It is likely that the nutrient profile in decoupled aquaponics will always differ slightly from a recommended nutrient solution for hydroponic plant production. As shown in this study, disadvantages for aquaponic plant production are not expected.

### 4.4 Abiotic and microbiological features of the nutrient solution

The less stable pH in the nutrient solution of the control (Fig 2) may be a result of the higher ratio of NH_4_-N (Table 4). It is known, that the uptake of NH_4_-N is related to an excretion of protons by the plant roots [36], whereby the nutrient solution becomes acidic. The addition of NaOH was effective in increasing the pH to reach target values without adding further nutrients (NPK) except of Na. A slight but continuously increasing EC value in the nutrient solution during the eight weeks of experiment, as detected in all treatments, is commonly reported as a result of accumulation of Na and Cl and macronutrients like Ca, Mg, K [42], and sulphate [43].

As expected, the TOC and DOC content were significantly increased by using fish water to mix the nutrient solution. DOC in the control was in a range with already reported values by Waechter-Kristensen, Caspersen [44]. However, as already mentioned, it seems that the increased TOC/DOC content in the aquaponics treatments did not have a positive effect on plant growth here.

The disinfection of the fish water based nutrient solution was considered to be successful because no CFU were detected in AP*dis* after disinfection (Fig 3). Nevertheless, CFU although commonly used, is just an estimate of the most probable number of bacteria as several aquatic microbes are not cultivable in standard media [45].

Generally only 1% of the waterborne microorganisms in ground and drinking water are cultivable *in vitro* [46]. But a thermal treatment of > 90°C for more than 15 min is an accepted procedure for disinfection [47] and additionally the temperature of the nutrient solution was maintained > 70°C for more than 45 min. Two days after successful disinfection lettuce plants were planted and the nutrient flow started. After the first five days after disinfection (on 21^st^ March 2016) the CFU had increased rapidly to around 2.1 x 10^4^ (AP*unt*) and 2.3 x 10^4^ (AP*dis*) units mL^-1^ in both aquaponic treatments. Compared to the reported CFU in RAS, rearing marine see bass (*Dicentrarchus labrax*) [48], the total content of CFU in fish water was relatively low in the present study. However, the microbial community and its density depends on fish species and varies from RAS to RAS [49]. As stated earlier, CFU is an estimate of the bacteria present in the nutrient solution; it is not reflecting the total abundance of microorganisms, since most of these organisms are not cultivable [47]. The reason for the high number of CFU might be the infection of the nutrient solution by spores or external microorganisms from the air and from the roots. The roots and its environment for instance, can be densely populated by microorganisms [44] which are easily spread within the water body. Therefore, disinfection will soon allow the establishing of a new microbiota. The increase of the CFU within the first five days after planting was less strong in the control compared to both aquaponics treatments and resulted in a significant lower content at the first measurement, suggesting suboptimal conditions for bacteria (i.e. heterotrophic bacteria) compared to the aquaponic groups. Usually organic matter (DOC and TOC) is tightly correlated with bacterial growth. Most probably, lower DOC/TOC concentrations might have restricted growth in the control. Nevertheless, in all three treatments a classical growth pattern (lag-, log-, stationary- and death phase) was observed. Still the lag- and the stationary phase were not so pronounced in our experiments which could be explained by the weekly sampling intervals for CFU [50]. At the end of the experiment the CFU ranged between 502 (AP*unt*) and 949 (AP*dis*) in all treatments as it was similarly reported for sterilized and non-sterilized aquaponic lettuce production by Pantanella, Cardarelli [51].

### 4.5 Effect on quality parameters of lettuce

#### 4.5.1 Phenolic compounds in lettuce leaves

It is widely recognised that plant-derived phenolic compounds and their antioxidant activity have beneficial health effects to humans [52]. Thus, phenolic compounds represent a commonly accepted quality parameter for the evaluation of plants. Phenolic compounds are ubiquitous in plants. In humans, they are mainly ingested via fruits and beverages. Vegetables are an important polyphenol source in the human diet [53]. As such, the intake of antioxidant-rich fruit and vegetables correlates inversely with the incidence of cancer [54]. Lettuce itself showed strong antioxidant and anti-inflammatory activities in *in vitro* studies [55]. However, the content of polyphenols is influenced by different abiotic factors such as e.g. light, temperature, water management, carbon dioxide concentration, and fertilisation [56]. The composition of phenolic compounds determined in the present study were already described for green lettuce and within the reported levels by DuPont, Mondin [57], Llorach, Martínez-Sánchez [58], and Ribas-Agustí, Gratacós-Cubarsí [59]. Applying different nutrient solutions as in the present study did not result in significant differences. However, the nutrient composition in the soil can have an effect on the content of phenolic acids and flavonoids [60–62]. An increasing N content, for instance, decreases the content of phenolic acids and flavonoids in different plant species [60, 61], whereas an increasing K content results in increasing phenolic compounds [61]. Both, N and K did not differ in the nutrient solution used for the different treatments in the present study. Here, the biggest differences were detected for P and S (Table 4). It was found by Radi, Mahrouz [61] that P fertilizer did not have a strong effect on phenolic compounds in comparison to N and K. S, however, mainly effects S containing secondary metabolites like glucosinolates [63], which is generally found in *Brassica* species. Furthermore, it has been reported that high molecular humic substances can stimulate the phenylpropanoid metabolism resulting in an accumulation of specific phenolics in maize (*Zea mays*) [64]. However, the use of different nutrient solutions with its partly varying nutrient concentrations (Table 4), different TOC/DOC content (Table 5), and a probably different bacterial composition, as used in the present study did not affect the analysed phenolic acids and flavonoids (Table 7).

#### 4.5.2 Nitrate content in lettuce leaves

The nitrate concentration is an important quality parameter for lettuce. It can have negative effects to human health when it is converted to nitrite by microorganisms [65]. In Germany a maximum level of 4000 (harvest from 01.10. until 31.03.) to 5000 mg NO_3_ kg^-1^ FW (harvest from 01.04. until 30.09.) is accredited [66]. Thus, the lettuce in the present study contained more nitrate (5502.1 to 5896.3 mg NO_3_ kg^-1^ FW) than accredited by Verordnung (EU) Nr. 1258/2011 [66]. Nevertheless, concentrations up to 4500 and 5406 mg NO_3_ kg^-1^ FW were also reported from other authors for fresh lettuce [67, 68]. The nitrate content of lettuce grown in hydroponic can be reduced by a combination of nitrate and ammonium nutrition [69]. As such, in contrast to the results in the present study it was expected that the higher ammonium content in the control resulted in a reduced nitrate content. One reason that the higher NH_4_-N content had no effect on the nitrate content in the leaves might be that the ammonium concentration was too low to influence the nitrate content significantly. Demsar and Osvald [69], for instance, found a reduced nitrate content of 13% by feeding at a NO_3_:NH_4_ ratio of 6.6:1. In the present study, the content of ammonium was much lower (13.7:1) and equal to other control treatments as reported by Gunes, Aktas [70]. Another reason for similar nitrate content in the control and the aquaponic treatments might be assigned to the higher amount of DOC in aquaponics compensating the ammonium effect in the control. As such, humic acid can reduce the accumulation of nitrate of diverse vegetable crops [71]. Furthermore, the nitrate content depends on growth period [72] and nitrate availability for plants [72–74]. Economakis and Koleilat [72] found out, that the longer lettuce is feed with nitrate, the more it accumulates in the plants. An earlier harvest in the present study with a fresh weight below 300 g could have resulted in lower nitrate content. Additionally, the nitrogen concentration in the nutrient solution could have been adjusted. Probably, a lower nitrogen concentration [75] would have resulted in a lower nitrate concentration without affecting the yield [72].

## 5 Conclusion

The benefits of nutrient and water recycling caused by a combined production of fish and plants in a decoupled aquaponic approach were clearly shown in this study. The recycling of fish water resulted in a reduction of fertilizer application of nearly 63% and a complete substitution of freshwater. No significant difference in growth of green open butterhead lettuce was observed, which is probably based on the same nutrient supply.

The content of DOC/TOC as well as microorganisms derived from RAS had no obvious positive/beneficial effects on the growth and phenolic compounds of lettuce.

The post adjustment of the nutrient profile using fish water for professional hydroponic application is more challenging as in conventional hydroponics (with rain water or tap water) but it was demonstrated that for most nutrients the set points were reasonable close to the recommended nutrient concentrations. With ongoing professionalization and standardisation of practises in decoupled aquaponic technology it is assumable that optimal nutrient profiles in decoupled aquaponic applications will be met with reasonable effort and comparable yields to conventional hydroponic production.

Additionally, butterhead lettuce (Salanova) has been proven to be a promising plant for potential intercropping as a further cash crop during the unproductive period for tomatoes in temperate regions, namely the winter.

## Supporting information

S1 Table Data used for the calculation of CO_2_-equivalents. (DOCX)

S1 File Nitrogen analysis of process waters and nutrient solutions. (DOCX)

S2 File Nutrient analysis of process waters and nutrient solutions. (DOCX)

S3 File Mineral elements in lettuce leaves. (DOCX)

S4 File Qualitative and quantitative analysis of phenolic compounds. (DOCX)
