# Supplementary material for: Lettuce (Lactuca sativa, variety Salanova) production in decoupled aquaponic systems: Same yield and similar quality as in conventional hydroponic systems but drastically reduced greenhouse gas emissions by saving inorganic fertilizer
Source: PLoS One. 2019 Jun 20;14(6):e0218368. doi: 10.1371/journal.pone.0218368 (PMC6586398; doi:10.1371/journal.pone.0218368)
Supplement: S1 Table — (DOCX) [file pone.0218368.s001.docx]

***S1 Table***

**Data used for the calculation of CO_2_-equivalents**

| Fertilizer | Nutrient content of source (%) |
| --- | --- |
| CAN | 15% N, 22% Ca |
| KNO_3_ | 13% N, 38% K |
| MgSO_4_ |  |
| KH_2_PO_4_ | 23% P, 28% K |

| Element | CO_2-e_ per nutrient based fertilizer (g g^-1^) |
| --- | --- |
| N | 7.6 |
| P | 1.26 |
| K | 1.2 |
| Ca | 0.311 |
| MgSO_4_ (total fertilizer) | 0.295 |

Example for calculation:

117 g CAN per L stock solution were used (control). 117 g CAN contains 17.6 g N and 25.7 g Ca. 17.6 g N result in 133.3 g CO_2-e_ per (7.6 g g^-1^)_,_ and 25.7 g Ca in 8 g CO_2-e_ (0.311 g g^-1^). In total the CO_2-e_ for CAN in the control is 141.4 g.
