# Supplementary material for: Lettuce (Lactuca sativa, variety Salanova) production in decoupled aquaponic systems: Same yield and similar quality as in conventional hydroponic systems but drastically reduced greenhouse gas emissions by saving inorganic fertilizer
Source: PLoS One. 2019 Jun 20;14(6):e0218368. doi: 10.1371/journal.pone.0218368 (PMC6586398; doi:10.1371/journal.pone.0218368)
Supplement: S1 File — (DOCX) [file pone.0218368.s002.docx]

***S1***

**Nitrogen analysis of process waters and nutrient solutions**

To determine the nitrogen content the samples were filtrated and analysed using continuous flow analysis (CFA; San++, Fa. SKALAR; Breda, The Netherlands). The used method is exactly describes by Suhl et al. (2016). As reference solution 0.584 mg L^-1^ ammonium standard solution (Merck KGaA; Darmstadt, Germany) and 0.565 mg L^-1^ nitrate standard solution (Merck KGaA; Darmstadt, Germany) were used. Dissolved ammonium nitrate was used to establish the standard series for CFA as following concentrations: 0.25 mg L^−1^, 0.5 mg L^−1^, 1.0 mg L^−1^, 2.0 mg L^−1^ and 3.0 mg L^−1^. Calcium chloride was used as rinse solution for the automatic sampler. The following working solutions for ammonium nitrogen (NH_4_-N) were used: buffer solution (66 g potassium sodium tatrate + 48 g sodium citrate trihydrate + 6 mL Brij (30%) in 2 L distilled (DI) water), sodium salicylate (12.5 g NaOH + 40 g sodium salicylate in 0.5 L DI water), sodium nitroprusside (0.5 g in 0.5 L DI water) and sodium dichloroisocyanurate (1.0 g in 0.5 L DI water). The measurement of NH_4_-N was performed at a wavelength of 660 nm. For nitrate nitrogen (NO_3_-N) analysis, a buffer solution with 3.405 g imidazole diluted in HCl and 3 mL Brij (30%) diluted in 1 L DI water were blended with a staining reagent with 50 mL H_3_PO_4_, 5 g sulfanilamide and 0.25 g naphthylethylendiamindihydrochloride diluted in 0.5 L DI water. The measurement was performed at a wavelength of 540 nm.

The different nitrogen forms were analysed in duplicate and expressed as mg L^-1^. The nitrogen forms NH_4_-N and NO_3_-N were additional summarized to mineral N (N_min_).

The respective microelements in the digestion solution were analysed at following wavelengths: Fe = 259.9 nm, Mn = 257.6 nm, Mo = 202.0 nm, Zn = 206.2 nm, Cu = 324.8, and B = 249.8 nm.

The different nitrogen forms and the mineral elements were analysed in duplicate and expressed as mg L^-1^. Both nitrogen forms NH_4_-N and NO_3_-N were summarized as mineral N (N_min_).
