# Supplementary material for: Lettuce (Lactuca sativa, variety Salanova) production in decoupled aquaponic systems: Same yield and similar quality as in conventional hydroponic systems but drastically reduced greenhouse gas emissions by saving inorganic fertilizer
Source: PLoS One. 2019 Jun 20;14(6):e0218368. doi: 10.1371/journal.pone.0218368 (PMC6586398; doi:10.1371/journal.pone.0218368)
Supplement: S2 File — (DOCX) [file pone.0218368.s003.docx]

***S2***

**Nutrient analysis of process waters and nutrient solutions**

The makro- (P, K, Ca, Mg, S, Na) and micronutrients (Fe, Mn, Mo, Zn, Cu, B) in the process waters and nutrient solutions were analysed using inductively coupled plasma-optical emission spectrometry ((ICP-OES), iCAP 6300Duo MFC, Fa. Thermo; Waltham, USA). After filtration, around 15 mL were firstly used to flush the ICP-OES, and 45 mL were following used for measurement. The following reference solutions were used to assemble the calibration curve for ICP-OES: blank 1.4 molar HNO_3_; 0–100 mg L^−1^ for P, Mg and Na, 0–300 mg L^−1^ for K and Ca, , 0–50 mg S L^−1^, and 0–5 mg L^-1^ for Fe, Zn, Mn, Mo, Cu, and B. The respective elements in the digestion solution were analysed at following wavelengths: P = 213.62 nm, K = 766.5 nm, Ca = 317.9 nm; Mg = 279.1 nm; S = 182.0 nm, Na = 589.6 nm, Fe = 259.9 nm, Mn = 257.6 nm, Mo = 202.0 nm , Zn = 206.2 nm, Cu = 324.8, and B = 249.8 nm.

The different mineral elements were analysed in duplicate and expressed as mg L^-1^. The nitrogen forms NH_4_-N and NO_3_-N were additional summarized to mineral N (N_min_).
