# Supplementary material for: Lettuce (Lactuca sativa, variety Salanova) production in decoupled aquaponic systems: Same yield and similar quality as in conventional hydroponic systems but drastically reduced greenhouse gas emissions by saving inorganic fertilizer
Source: PLoS One. 2019 Jun 20;14(6):e0218368. doi: 10.1371/journal.pone.0218368 (PMC6586398; doi:10.1371/journal.pone.0218368)
Supplement: S3 File — (DOCX) [file pone.0218368.s004.docx]

***S3***

**Mineral elements in lettuce leaves**

For analysing mineral elements in lettuce leaves, the freeze dried samples were analysed by ICP-OES. In detail, for sample preparation, between 0.498 and 0.502 gram of the sample material was weight into special, de-ionised vessels. The vessels were placed into a microwave (MARS Xpress, CEM; North Carolina, USA) after the addition of 5 mL nitric acid (HNO_3_, 65%) and 3 mL hydrogen peroxide (H_2_O_2_, 30%). The digestion was carried according to following program: 1^st^ step = 20 minutes (min.) to reach 200 °C, 2^nd^ step = 5 min. at 200 °C, 3^rd^ step = 1 min. to reach 210 °C, 4^th^ step = 5 min. at 210 °C, 5^th^ step = 1 min. to reach 220 °C, 6^th^ step = 5 min. at 220 °C and 7^th^ step = 30 min. to cool down to room temperature. The resultant solutions were transferred into 50 mL volumetric flasks using DI water. After the samples were filtered into plastic flasks the analysis of the mineral elements followed using ICP-OES (iCAP 6300 Duo MFC, Fa. Thermo; Waltham, USA). The operating power of the ICP-OES was 1150 W RF and the nebulizer gas flow 0.55 L min^-1^. The carrier gas and plasmogen was argon and the cross-flow nebulizer (MIRA Mist; Thermo Scientific, Cambridge, UK) were used for performance for axial (Fe) and radial (P, K, Ca, Mg), view. To prepare a reference solutions for each element, a single-element solution were prepared in 1.4 mol L^-1^ HNO_3_ and the calibration curves were created with following reference solutions: blank 1.4 mol L^-1^ HNO_3_; 0–50 mg L^-1^ of P and Mg, and 0–10 mg L^-1^ of Na, 0–400 mg L^-1^ of K; 0–100 mg L^-1^ of Ca, 0–20 mg L^-1^ of S, 0–5 mg L^-1^ of Fe and Zn, and 0–1 mg L^-1^ of Mn, Mo, and B. The respective elements in the digestion solution were analysed at wavelengths describes in 2.2.1. The backup ensued by double determination and the results were expressed as mg 100 g^-1^ FW.
