# Supplementary material for: Lettuce (Lactuca sativa, variety Salanova) production in decoupled aquaponic systems: Same yield and similar quality as in conventional hydroponic systems but drastically reduced greenhouse gas emissions by saving inorganic fertilizer
Source: PLoS One. 2019 Jun 20;14(6):e0218368. doi: 10.1371/journal.pone.0218368 (PMC6586398; doi:10.1371/journal.pone.0218368)
Supplement: S4 File — (DOCX) [file pone.0218368.s005.docx]

***S4***

**Qualitative and quantitative analysis of phenolic compounds**

The compounds were separated at 35 °C and a flow rate of 0.4 mL min.^-1^ using a 150 x 2.1 mm, 3µm, C16 column (AcclaimPA, Thermo Fisher Scientific; Waltham MA, USA). For analysis, eluent A (0.5% formic acid in water, Merck; Darmstadt, Germany) and eluent B (40% Acetonitril, J.T. Baker; Deventer, The Netherlands) were used. As such, following eluent gradient was used: 0-1 min. 0.5% B, 1-10 min. 05.-40% B, 10-12 min. 40% B, 12-18 min. 40-80% B, 18-20 min. 80% B, 20-24 min. 80-99% B, 24-30 min. 99-100% B, 30-34 min. 100-0.5% B, and 34-39 min. 0.5% B.
